# Ant workers produce males in queenless parts of multi-nest colonies

**DOI:** 10.1038/s41598-020-58830-w

**Published:** 2020-02-07

**Authors:** Julia Giehr, Lisa Senninger, Katja Ruhland, Jürgen Heinze

**Affiliations:** University of Regensburg, Zoology/Evolutionary Biology, Universitätsstr. 31, D-93053 Regensburg, Germany

**Keywords:** Evolutionary theory, Social evolution

## Abstract

Workers of several social insects are capable of gaining direct fitness by laying unfertilized eggs, which then develop into males. However, under queenright conditions, direct reproduction of workers is usually prevented by queen-induced regulatory mechanisms. In nature, some ant colonies inhabit multiple nests sites (polydomy). This might allow workers to escape queen control and to reproduce. However, whether worker-produced brood survives after colony reunion in seasonally polydomous species remains unclear. In several species, worker-produced eggs and male-destined larvae are selectively destroyed in queenright colonies. Here, we test whether workers discriminate between queen- and worker-produced larvae during colony reunion. We examined the reproductive success of workers in queenless subcolonies of our study species *Temnothorax crassispinus*. Our results show that present brood did not inhibit worker reproduction but had a positive effect on worker lifespan. Larvae produced by workers were readily integrated into queenright subcolonies during colony reunion and these larvae successfully developed into adult males.

## Introduction

Hamilton’s theory of inclusive fitness elegantly explains the altruistic behavior of workers in social wasps, bees, and ants by gains in indirect fitness^1^. Through helping a related egg-layer, workers can greatly increase the relative’s reproductive output and in this way can transmit more copies of their genes indirectly than directly by producing own offspring. In addition to indirect fitness benefits, workers might also attempt to obtain direct fitness. In most species of social Hymenoptera, workers are capable of laying at least unfertilized eggs, which can develop into males. Worker reproduction is usually impaired in the presence of a queen through its pheromones or aggressive policing^2–4^, but in queenless colonies workers may readily produce sons^5,6^.

Colonies of several ant species seasonally inhabit multiple nest sites, probably because of spatial constraints or because it allows them to forage more efficiently and to better exploit and defend a territory (polydomy^7,8^). In addition, polydomy might allow workers to escape queen control^9–11^. Indeed, sex ratios are often more male-biased in multi-nest than in single-nest colonies^12,13^. However, polydomy is often associated with the presence of multiple queens and colony founding by budding, which both also might lead to male bias^7^. Furthermore, whether worker-produced brood survives when colony fragments of a seasonally polydomous species fuse for hibernation remains unclear. Workers of several ants and wasps are known to discriminate at least between queen- and worker-produced eggs and eat the latter^14–16^, and male brood is selectively destroyed regardless of origin in other species^17,18^. However, previous studies in *Temnothorax unifasciatus* ants revealed that manually introduced queen-or worker produced eggs had been integrated independent of origin in queenless and queenright subcolonies^19^. Nevertheless, it remains unclear whether the reunion of reproductive workers with the queenright fragment affects the survival of the brood.

Here we examine, whether polydomy can increase worker direct fitness in the monogynous ant *Temnothorax crassispinus*^20^. Colonies of *Temnothorax* nest in spatially limited cavities in rock cracks, rotting branches, or hollow acorns, and in summer often space out to multiple nests^21–28^. In queenless conditions, *Temnothorax* workers form rank orders by antennal boxing and biting and only the highest ranking workers lay unfertilized, male-destined eggs^19,29–32^. Genetic analyses revealed that in both queenless and queenright colonies of *T. crassispinus* about 20% of the males are not offspring of the queen^33,34^. We split colonies into a queenright and queenless subcolony for ten weeks and then allowed the subcolonies to reunite again. We investigated, whether worker-produced male larvae from queenless subcolonies are accepted by workers from the queenright colonies during and after colony fusion. We compared the reproductive output of queenless and queenright subcolonies and checked for possible inhibitory effects of queen-produced larvae on worker egg laying and for discrimination against larvae from the queenless subcolony.

The results of our study show that *T. crassispinus* workers successfully reproduce under queenless conditions. The presence of brood from the original colony delays but does not inhibit worker reproduction in queenless subcolonies but in contrast might have a beneficial effect on worker survival. Workers from queenright subcolonies accept adult workers and larvae from the queenless subcolony and worker-produced larvae were reared to adulthood. Behavioral observations show that workers preferentially cared for brood items that originated from their own subcolony, but queenless and queenright larvae were equally cared for.

## Results

### Brood development in queenright and queenless subcolonies

**Figure 1:**
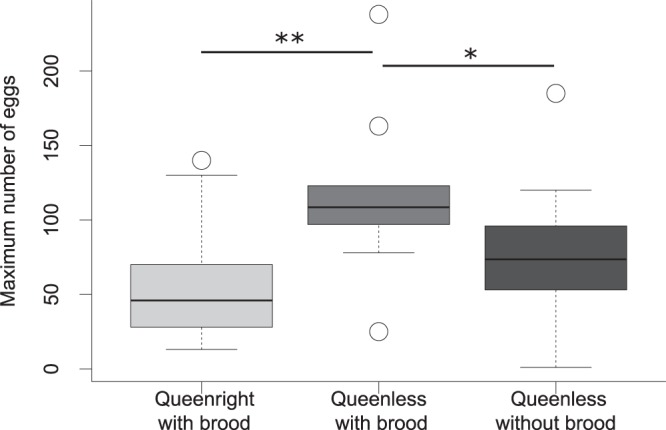
Maximum egg number in queenright and queenless subcolonies of the ant *Temnothorax crassispinus* with brood and in queenless subcolonies without brood. Queenless subcolonies that had received brood items from the stock colony at the beginning of the experiment produced more eggs than subcolonies from the other two groups. Boxplots show medians, 25 and 75 quartiles, and 95% percentiles (*p < 0.05 **p < 0.01 corrected for a false discovery rate according to Benjamini and Hochberg^69^).

First eggs were produced five to six weeks after colony splitting. The treatment groups (1) queenright, 2) queenless with queen-derived larvae and 3) queenless without queen-derived larvae) differed in the onset of reproduction (Kruskal-Wallis test: Χ² = 20.70, df = 2, p < 0.0001; for details see Table 1). Queenless subcolonies without brood laid their first eggs on average one week earlier than queenright subcolonies (p < 0.0001) or queenless subcolonies with larvae (p = 0.008). Subcolonies reached their reproductive peak (highest egg number inside the nest) after seven to eight weeks (Kruskal-Wallis test: Χ² = 1.59, df = 2, p = 0.452). Furthermore, queenless subcolonies with brood reached higher maximal numbers of eggs than colonies without brood and contained more than twice as many eggs as queenright colonies (Kruskal-Wallis test: Χ² = 12.31, df = 2, p = 0.002, QL with brood vs. QL: p = 0.019, QL with brood vs. QR p = 0.002, QR vs. QL: p = 0.169, Fig. 1). The developmental time from egg to larva was extended by approximately two to three weeks in queenless compared to queenright subcolonies (Kruskal-Wallis test: Χ² = 21.62, df = 2, p < 0.0001, QL with brood vs. QL: p = 0.806, QL with brood vs. QR p = 0.0008, QR vs. QL: p = 0.0002) but the eggs developed equally well into larvae (ratio eggs and larvae two weeks later: Kruskal-Wallis test: Χ² = 4.90, df = 2, p = 0.086). The maximum number of larvae produced within ten weeks did not differ among the three groups (Kruskal-Wallis test: Χ² = 3.90, df = 2, p = 0.142).

**Table 1:** Measures of reproductive performance in queenright and queenless (with and without brood) subcolonies of the ant *Temnothorax crassispinus*.

(median, Q1, Q3)	Queenright n = 30	Queenless with brood n = 10	Queenless without brood n = 20
weeks to first egg	6.0, 6.0, 6.9	6.0, 5.0, 6.0	5.0, 5.0, 5.0
time until reproductive peak (weeks)	8.0, 6.0, 8.0	7.0, 7.0, 7.8	7.0, 6.0, 7.0
maximum egg number	46.0, 28.5, 70.0	108.5, 97.25, 120.75	71.5, 44.0, 92.25
weeks to first larvae	3.0, 2.0, 3.0	5.0, 4.0, 5.0	5.0, 4.0, 5.0
relation larvae to eggs after two weeks (%)	82.0, 55.1, 115.0	46.8, 37.3, 62.6	73.5, 49.2, 91.3
maximum larvae number	77.0, 57.0, 98.5	112.50, 84.8, 149.0	78.5, 42.8, 115.5

Values shown are median, first quartile (Q1) and third quartile (Q3).

### The absence of brood items decreased worker survival

**Figure 2:**
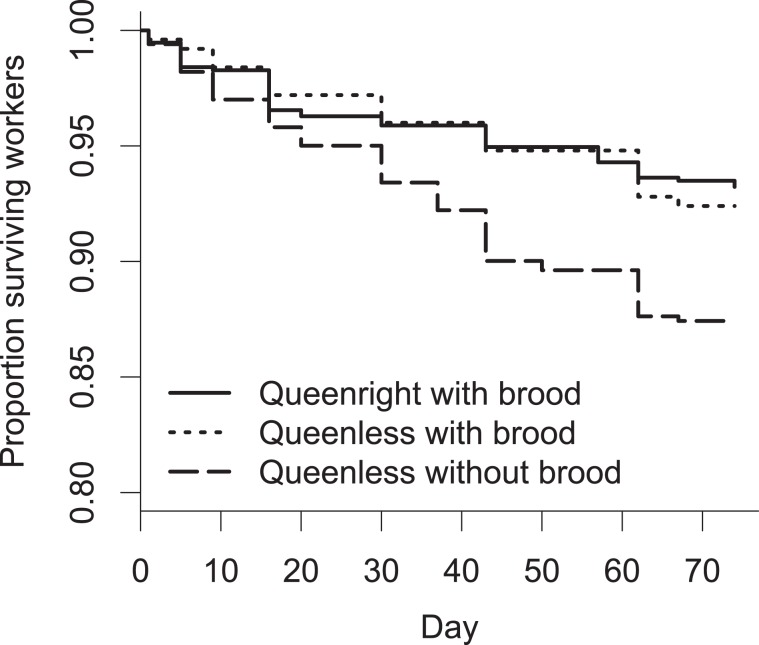
Survival of *T. crassispinus* workers in queenright (Queenright with brood) subcolonies and subcolonies that had received brood from the stock colony (Queenless with brood) or not (Queenless without brood). Significantly more workers died in subcolonies that had received larvae at the beginning of the experiment in queenless subcolonies without brood and queenright subcolonies.

Worker survival differed significantly among the treatment groups (Kaplan-Meier survival analysis: Χ² = 13.2, df = 2, p = 0.001, Fig. 2). Survival was significantly decreased in queenless subcolonies that had not received brood items (dead workers: 5, 1.25, 6) compared to queenright colonies (dead workers median, Q1, Q3: 2.5, 1, 5; p = 0.0015) and probably also queenless subcolonies with brood (dead workers: 1, 0, 2.75; p = 0.057, queenright vs. queenless with brood: p = 0.627).

### Workers did not selectively reject queen- or worker-produced larvae during colony reunion

The three groups did not differ in the number of larvae before we allowed the subcolonies to reunite after ten weeks (Kruskal-Wallis test: Χ² = 4.82, df = 2, p = 0.090, for details see Table 2). When placed into a common arena, 13 of the 17 colonies reunited within 29 hours (mean ± s.d. 20.8 ± 8.6). Four pairs of subcolonies did not reunite within the observation period. Eight of the colonies fused in the queenright nest and five reunited in the queenless nest. In nine observed cases (five colonies), workers transported larvae from their own subcolony to the new nest, and only in one observation, a worker carried a larva from the other subcolony. Adults were carried by members of the their own subcolony (three observations) as well as the other subcolony (three observations). We did not observe attacks of workers or killed workers within the seven-day observation period and did not find any difference in behavior or colony composition between parts that merged and those that did not.

**Table 2:** Changes in the number of larvae during seven days after the reunification of queenless and queenright subcolonies of the ant *Temnothorax crassispinus*.

	Before reunion	1^st^ day after reunion	7^th^ day after reunion	Change in the number of larvae
Reunited colonies (n = 13)				
Queenright	57, 30, 80	56, 28, 71	30, 26, 51	−12, −4, −33
Queenless	51, 38, 70	38, 30, 47	30, 28, 47	−12, −8, −27
Not reunited subcolonies (n = 4)				
Queenright	57, 32, 70	56, 30, 68	45, 40, 51	−8, −4, −19
Queenless	50, 36, 61	36, 29, 47	25, 24, 31	−9, −7, −24

The number of larvae did not decrease significantly in queenright or queenless colonies over a seven-day period and the mortality of larvae after seven days was similar for larvae originally produced in the queenright and queenless subcolony. Values are given as median, first quartile (Q1) and third quartile (Q3).

In the reunited colonies, larvae from queenright as well as queenless subcolonies were accepted after fusion, and there was no significant decrease in the number of either type of larvae during the seven days following fusion, during which the larvae from queenright and queenless subcolonies could still be distinguished because of their coloration (queenright larvae: 7th day after reunion, median Q1, Q3: 30, 26, 51; Friedman test before reunion, 1st day after reunion, 7th day after reunion: Χ² = 0.275, df = 2, p = 0.871; queenless larvae: 30, 28, 47; Χ² = 0.039, df = 2, p = 0.981). Similarly, in the colonies that refused to reunite there was no change of the number of larvae (queenright larvae 7th day median Q1, Q3: 45, 40, 51; Friedman test before reunion, 1st day after reunion, 7th day after reunion: Χ² = 0.50, df = 2, p = 0.779; queenless larvae: 25, 24, 31; Χ² = 1.29, df = 2, p = 0.526). There was also no significant difference in the decrease of numbers between red or blue larvae within the seven days after fusion (reunited colonies: Mann-Whitney U-test: W 73.5, p = 0.589, not reunited colonies: W = 9.5, p = 0.772). Workers in reunited colonies generally seemed to preferentially care for brood from their original subcolony (instances of carrying, grooming, and feeding observed during 120 min, Wilcoxon test: V = 66, p = 0.004, own larvae median, Q1, Q3: 14.0, 11.5, 24.0; alien larvae: 11.0, 7.5, 16.5). Larvae from formerly queenless and queenright subcolonies received equal attention (V = 29, p = 0.754, queenless larvae median, Q1, Q3: 14.0, 10.5, 19.5; queenright larvae: 11.0, 10.0, 20.5). This also indicates that color did not affect brood care.

### Colony reunion affects colony growth and offspring sex-ratio

**Figure 3:**
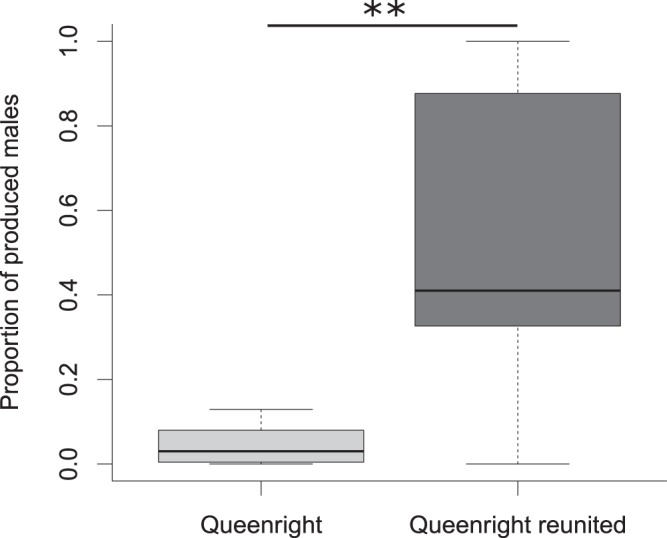
Proportion of produced males (males/all offspring) in still separated queenright subcolonies (“Queenright”) and reunited colonies (“Queenright reunited”). Queenright subcolonies that did not reunite with their queenless counterpart produced significantly more males than already reunited colonies. Boxplots show medians, 25 and 75 quartiles, and 95% percentiles (*p < 0.05 **p < 0.01 corrected for a false discovery rate according to Benjamini and Hochberg^69^).

Worker number differed between the colonies 16 months after colony reunion (Kruskal-Wallis test: Χ² = 13.258, df = 3, p = 0.004). At this time, queenright colonies that experimentally had been kept separate from queenless subcolonies had more workers (n = 8; worker number median, Q1, Q3: 36.5, 32, 71) than reunited colonies (n = 13; p = 0.03; worker number: 22, 5, 26), not reunited colonies (n = 4, p = 0.01, worker number: 10.5, 7.75, 14.75) or queenless subcolonies (n = 9; p = 0.009; worker number: 15, 12, 20; reunited colonies vs. not reunited subcolonies and separated queenless subcolonies p > 0.05). However, before the first prepupae developed and the males eclosed, the colonies did not differ in the number of larvae (Kruskal-Wallis test: Χ² = 5.21, df = 3, p = 0.157; median, Q1, Q3: reunited colonies: 38, 12, 67; not reunited colonies: 25.5, 22.25, 31.00; QR colonies: 69, 47, 127.25; QL colonies: 33, 22, 59). Subcolonies that had not reunited produced less offspring (males + workers; no female sexuals produced; N = 4; median, Q1, Q3: 5.5, 1.5, 9; Kruskal-Wallis test: Χ² = 10.66, df = 3, p = 0.014) than queenright colonies (N = 8: 46, 33.3, 81; p = 0.025) and queenless colonies (N = 9: 27, 16, 51; p = 0.025; reunited colonies N = 9: 39, 17, 51; p = 0.075). However, sex ratios were significantly more male-biased in reunited colonies than in still separated queenright subcolonies, which rarely produced males but focused on worker production (Mann-Whitney U-test: W = 7, p = 0.006; males/total offspring median, Q1, Q3: reunited colonies: 0.41, 0.33, 0.88; queenright: 0.03, 0.007, 0.08, Fig. 3; not reunited produced too few sexuals for a meaningful statistical analysis).

## Discussion

Seasonal polydomy has been suggested to allow workers to escape queen and worker policing and to thus achieve direct fitness by producing own male-destined larvae from unfertilized eggs^7,9^. However, whether worker-derived brood survives when the colonies fuse for joint hibernation remained unclear. Here we document that males produced by workers in queenless subcolonies survive colony reunion and lead to male-biased sex ratios in the seasonally polydomous, monogynous ant *Temnothorax crassispinus*.

Worker-produced larvae did not differentially disappear after the queenless and queenright subcolonies fused again, and reunited colonies reared a significantly higher percentage of males than queenright subcolonies, which had not reunited with their queenless counterpart. This suggests that larvae produced by workers in queenless subcolonies readily survive and are cared for in the presence of the queen and after hibernation contribute to the males reared by queenright colonies. Genetic data on the maternity of males from natural colonies corroborate this finding and suggest that workers of *T. crassispinus* obtain direct fitness^34^.

We did not find any evidence for queen-produced brood inhibiting worker reproduction, in contrast to what has been reported previously for honeybees^35,36^ and ants^37–39^. Instead, queenless subcolonies that contained larvae from the original colony, produced larger numbers of eggs than queenright subcolonies or queenless subcolonies without brood. This positive effect of larvae on worker egg laying matches observations by Smeeton^40^ and might suggest that larval inhibition depends on larval stage and brood/worker ratio. Similarly, we did not observe any negative effect of larvae on the life expectancy of workers, in contrast to what has been found in honey bees^41,42^. Workers that had to care for larvae appeared to live slightly longer than workers from colonies that initially contained no brood. Such workers might have spent more time inactively than workers, which had to engage in brood care. Inactivity may negatively affect worker survival^43^ (but see^44^). Furthermore, the presence of larvae may affect the nutritional balance of the colony. Workers in colonies with larvae generally consume more food with a higher protein to carbohydrate ratio to provide larvae with proteins^45^. Although workers can barely digest proteins directly, pre-digestion by larvae and the uptake of larval gland secretion may enable them to uptake protein-based nutrients, which might positively affect their nutritional status and lifespan^45–47^.

The absence of the queen appeared to delay egg development: eggs produced in queenless subcolonies needed more time before they hatched into larvae than eggs in queenright subcolonies. This is presumably not due to the ploidy of eggs, as the development times of haploid and diploid eggs in ants and other Hymenoptera appear to differ only marginally if at all^48–50^. The observed difference might rather stem from intra-colonial conflicts, in that egg eating associated with dominance behavior and worker policing might have caused a frequent exchange of newly produced eggs during the initial phase^19,29^.

In contrast to what has been shown for worker-laid eggs in other social insects^15,16,51^, our behavioral observations did not indicate a categorical rejection or destruction of worker-produced larvae following colony reunion (for *T. unifasciatus* see^19^). Reunited queenright colonies produced a significantly more male-biased sex ratio than queenright subcolonies, which had not fused with their queenless counterpart. This matches observations in natural queenright colonies^28^ and indicates that worker-produced larvae survived colony fusion and hibernation.

Workers preferentially interacted with larvae that had been produced in their original subcolony. This suggests that workers are capable of recognizing the origin of the larvae. Environmental cues are important in nestmate discrimination in *T. crassispinus*^52^. In our experiment, subcolonies were kept under identical conditions and were treated equally, except for the addition of different food colorants. Although food dyes do not appear to affect egg treatment^19^, egg staining may have changed larval odor and workers might have preferred to care for larvae with a familiar scent. Alternatively, during colony fusion, workers from the two subcolonies may have settled in and become faithful to different areas in the nest. Such spatial fidelity has previously been observed in other species of the genus^53^.

In the long run, colony fusion with reproductive workers appears to have a negative impact on colony fitness, as reunited colonies contained significantly fewer workers after one year than still separated queenright colonies. Although workers did not attack others during reunification (see also^19^), we cannot completely rule out later aggression among workers associated with reproductive status. Long-term conflict might have had negative effects on worker survival and colony growth. Furthermore, stress and changing colony structures might lead to task shifts in workers^54^. Task allocation is highly flexible in social insect workers and shifts can cause physiological changes, which again affect worker lifespan^54–57^. However, under natural conditions, *T. crassispinus* workers can migrate between nests^33,34,52^, which might enable them to avoid conflict and negative effects of long-term colony fusions. Furthermore, frequent intercolonial fusions and queen usurpation^23,33,34,58^ might outbalance possible energetic costs resulting from worker reproduction^59,60^.

In summary, our data show that *Temnothorax crassispinus* workers are capable of gaining direct fitness in queenless nests of polydomous colonies. Queenless subcolonies were highly productive. Workers from queenless subcolonies were not prevented from entering the queenright nest and could bring their larvae into the joint nest. After colony reunion, workers appeared to be capable of differentiating between the brood items but all brood items were equally cared for. Surprisingly, queenright subcolonies rarely produced males, indicating that workers might contribute to male production also in nature.

## Material and Methods

*Temnothorax crassispinus* is a small monogynous, monandrous ant species (a single, singly-mated queen per colony), which lives in small colonies of up to 300 individuals in hollow acorns or twigs throughout Eastern Central Europe^28,31,61,62^. Previous field studies had suggested that *T. crassispinus* is seasonally polydomous^28^, similar to its sibling species *T. nylanderi*^23,63^. Colonies were collected in August 2017 in deciduous forests around Regensburg, Germany. From 30 colonies we set up 30 queenright (QR) and queenless (QL) subcolonies each, resulting in 60 subcolonies (for a schematic figure see Supplementary S2). All 30 queenright subcolonies and 10 of the queenless subcolonies received 25 workers and 25 larvae of all developmental stages from the initial colony. The remaining 20 queenless subcolonies consisted of 25 workers without larvae to investigate a possible influence of brood from the original colony on reproduction as reported, e.g., for *Novomessor* ants^37^. To be later capable of distinguishing between workers from queenright and queenless subcolonies, workers in queenright colonies were marked by clipping the right tarsae of the middle leg, workers of queenless colonies by clipping the left tarsae.

Each subcolony was reared in a separate box (9.6 cm × 9.6 cm × 3 cm) containing a nest composed of a plastic frame sandwiched between two microscope slides (1.2 cm × 5 cm × 0.3 cm) with a narrow entrance (0.3 cm × 1 cm × 0.3 cm). The ants were fed twice per week with cockroaches and honey. Honey was colored with commercially available food dyes to allow to distinguish between larvae reared in queenless and queenright subcolonies (red: Allura red AC, E129, 12.5% pure color, 2% aluminum; blue: Brilliant blue FCF, E133, 9.26% pure color, 3.6% aluminum, carrying agent sulfate/chloride, RBV Birkmann GmbH & Co; 4 g per liter of solution). Red and blue food coloring were used for queenright and queenless subcolonies, respectively, as in previous experiments^64,65^. The color of food dyes did not have an influence on brood care or larval survival rate (^19^ and see results).

Subcolonies were kept in incubators at artificial summer conditions (12 h 26 °C/12 h 23 °C day/night cycle) to support egg laying and after five weeks was gradually reduced to 18/13 °C (2 °C every three weeks for nine weeks), to simulate pre-hibernation conditions and facilitate the natural merging of experimental colonies before hibernation (see^26,27^). Workers and brood items were counted once per week to monitor colony productivity and survival.

The queenright and queenless subcolonies from 17 of 30 stock colonies were transferred after ten weeks into a larger arena (nine queenless subcolonies, which initially received queen-deprived brood and eight queenless subcolonies that did not receive brood items at the beginning; 13 pairs of subcolonies were not allowed to fuse and used for later experiments). The arena (diameter 13.5 cm) contained uncolored honey, cockroaches/*Drosophila* flies, and water. We placed the nests at the opposite sides of the arena and observed the arena for 20 minutes. After the initial observation phase fused colonies were observed for 10 minutes every hour on the first day (total 60 minutes per colony) and 10 minutes every two hours on the second, third and fourth day (40 minutes per day per colony). We measured the time until reunification of the subcolonies, i.e., until workers had completely moved together, and noted interactions among workers and with brood items. Non-antagonistic interactions with brood items (feeding, grooming and carrying inside the nest) were counted as brood care. Observations were conducted blindly, i.e., the observing person did not have information about the experimental setup, the meaning of the colored food, or the origin of the marked individuals.

The staining of the larvae vanishes quickly when workers were fed with uncolored honey in the shared arena, thus items and workers could be counted for a maximum of seven days. Subsequently, temperature was gradually decreased to 6 °C/2 °C during the following six weeks to provide hibernating conditions. The males needed in total 19 months to develop from eggs to adults and eclosed after the second hibernation (3.5 months, minimum 6 °C/2 °C). This might come from the late start of egg production at the end of the natural reproductive season (middle of October) and the shortened hibernation period (2 months). In total, colonies were kept for more than 19 months to monitor worker and offspring development. Worker and brood number decreased strongly in four reunited colonies (less than five workers and/or less than ten larvae) within 19 months and these colonies had to be excluded from the analyses of male offspring. One queen died during hibernation and the colony was excluded from the analyses of brood items and offspring numbers after 16 months.

Data are given as median, first quartile (Q1) and third quartile (Q3) and were analyzed with R v. 3.2.3 software^66^. We used Kruskal-Wallis Chi²-tests for independent and Friedman tests and Wilcoxon tests for dependent data, as data were not normally distributed (Shapiro-Wilk test p < 0.05). Survival of the workers was compared with Kaplan-Meier survival analysis (“survival” package^67^) and survdiff pairwise comparisons (“survminer” package^68^) for group comparisons. We only included verifiably dead individuals in the survival analysis.

### Ethics approval

*Temnothorax crassispinus* is an unprotected ant species. All experiments comply with European laws.

## Supplementary information

Supplementary file S1.

Supplementary file S2.

## Data Availability

The datasets of the article are available in the Supplementary File S1.
