# Supplementary material for: Ant workers produce males in queenless parts of multi-nest colonies
Source: Sci Rep. 2020 Feb 7;10:2152. doi: 10.1038/s41598-020-58830-w (PMC7005753; doi:10.1038/s41598-020-58830-w)
Supplement: Supplementary file 2 — Supplementary file S2. [file 41598_2020_58830_MOESM2_ESM.pdf]

Electronic Supplemental Material S2

Ant workers produce males in queenless parts of multi-nest colonies

J. Giehr, L. Senninger, K. Ruhland and J. Heinze

University of Regensburg, Department of Zoology/Evolutionary Biology, D-93053 Regensburg

Corresponding author: Julia Giehr (Julia.Giehr@ur.de)

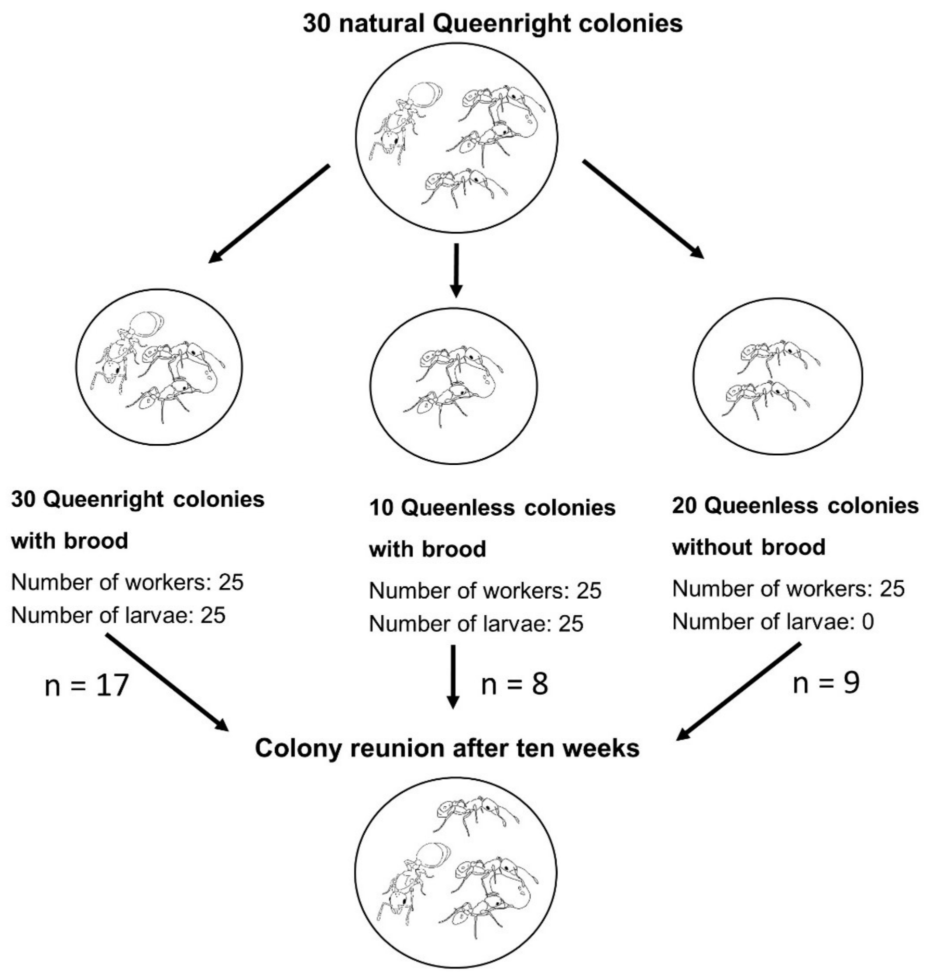

Figure S1, Schematic representation of the experimental set up. In total, 30 natural queenright colonies were splitted in 10 queenless colonies with queen-deprived brood and 20 queenless colonies without queen-deprived brood. All colonies were allowed to reproduce independently for five weeks. Colonies were reunited in a newly introduced arena five weeks later, when eggs developed successfully into larvae and showed a visible blue (queenless larvae) and red (queenright larvae) staining.
